# Partner Choice Patterns Among the Descendants of Turkish Immigrants in Europe

**DOI:** 10.1007/s10680-012-9265-2

**Published:** 2012-06-22

**Authors:** Doreen Huschek, Helga A. G. de Valk, Aart C. Liefbroer

**Affiliations:** 1 Phoolan Devi Institute, VU University Amsterdam, De Boelelaan 1105, 1081 HV Amsterdam, The Netherlands; 2 Netherlands Interdisciplinary Demographic Institute (NIDI), P.O. Box 11650, 2502 AR Den Haag, The Netherlands; 3 Interface Demography, Vrije Universiteit Brussels, Pleinlaan 2, 1050 Brussel, Belgium; 4 Department of Social Research Methodology, VU University Amsterdam, De Boelelaan 1081, 1081 HV Amsterdam, The Netherlands

**Keywords:** Second generation migrants, Intermarriage, Europe, Turks, Partner choice, Migrants de seconde génération, Mariage mixte, Europe, Turcs, Choix du partenaire

## Abstract

We examine the partner choice patterns of second-generation Turks in 13 European cities in seven countries. We not only compare intermarriage versus endogamous marriage, but also explicitly include the choice of a second-generation partner of the same origin and of a partner of other migrant origin as important alternatives. In Europe, populations are made up increasingly of migrants and their descendants resulting in new alternative partner options not open before. Findings suggest that second-generation Turks who choose a second-generation partner seem to be located between the partner choice of a first-generation and native partner in terms of family values and contact to non-coethnic peers. The choice of a partner of other migrant origin hardly differs in these characteristics from the choice of a native partner. Context variables such as group size and type of integration policies seem to play a role for the likelihood of having a first-generation versus a second-generation partner of Turkish origin but not for the likelihood of exogamous partner choice. A second-generation partner is the most popular choice in Germany but represents a minor option in the other countries. Furthermore, a partner of other migrant origin is more common among men but is in some countries more popular than a native partner among Turkish second-generation men and women.

## Résumé

Nous examinons les schémas de choix du partenaire pour les Turcs de seconde génération dans treize villes européennes de 7 différents pays. Nous ne nous limitons pas à comparer mariage mixte (entre un(e) partenaire d’origine turque et une(e) partenaire originaire du pays) versus mariage endogamique, mais nous incluons également le choix d’un partenaire de seconde génération de même origine ou d’un autre pays d’origine comme des alternatives importantes. En Europe, les populations incluent un nombre grandissant de migrants et de descendants de migrants, ce qui conduit à de nouvelles options possibles en termes de choix de partenaire. Les résultats suggèrent que les Turcs de seconde génération qui choisissent un partenaire de seconde génération se situent, en termes de valeurs familiales et contacts avec des pairs d’autres ethnies, entre ceux faisant le choix d’un partenaire de première génération et ceux choisissant un natif comme partenaire. En revanche, ces caractéristiques différent peu dans le cas de choix d’un partenaire d’une autre origine migratoire versus choix d’un partenaire originaire du pays. Des variables contextuelles telles que les effectifs du groupe et le type de politiques d’intégration semblent avoir un impact sur la probabilité d’avoir un partenaire de première génération versus un partenaire de seconde génération d’origine turque, mais pas sur celle d’un choix de partenaire exogame. Un partenaire de seconde génération est le choix le plus fréquent en Allemagne mais ne représente qu’une option minoritaire dans les autres pays. De plus, choisir une partenaire d‘une autre origine migratoire est plus fréquent chez les hommes, mais dans certains pays ce choix s’avère plus fréquent qu’une(e) partenaire originaire du pays tant pour les femmes que les hommes turcs de seconde génération.

## Introduction

The children of former labor migrants from Turkey constitute a growing proportion of the young adult population throughout Europe. As a result of their growing numerical presence, the family formation choices of the second generation are increasingly important for the demographic future of European societies (Coleman 2006; Lucassen and Laarman 2009), yet it is unclear what shape their family formation choices will take. On the one hand, they are influenced by ideas and behavioral patterns that are dominant in their settlement countries. On the other hand, their parents were socialized in the Turkish tradition of partner choice and family formation, which is characterized by early union formation and a strong familial influence on the union formation choices of their children (De Valk et al. 2004; Kagitçibasi 1996; Nauck 2002). The present study will explore this issue by studying the partner choices of second-generation Turks in seven European countries.

So far, research primarily focuses on whether migrants intermarry or choose a partner from their own group. Classical studies in the United States perceived intermarriage as the final step of integration (Gordon 1964; Lieberson and Waters 1988) and as an indicator for diminishing social and cultural boundaries between groups (Alba and Golden 1986; Kalmijn 1998). These studies generally assume that assimilation is a gradual process, and that with prolonged residence intermarriage rates will increase. Both North-American and European studies on intermarriage of first-generation migrants report low levels of intermarriage between groups of different origins (Alba and Nee 2003; Dribe and Lundh 2011; Furtado and Theodoropoulos 2011; Kalmijn and Van Tubergen 2010; Lee and Boyd 2008; Lucassen and Laarman 2009; Muttarak and Heath 2010; Sánchez-Domínguez et al. 2011). In addition, although an overall increase over generations in levels of intermarriage is found in both North America and Europe, differences between origin groups remain (e.g., Lee and Boyd 2008; Muttarak and Heath 2010). Earlier studies have reported that the second generation also continues to have spouses from their parents’ country of origin. This is believed to be associated with a tendency of migrants and their descendants to hold on to values and customs from their country of origin, which is reinforced by continued migration pressure from their country of origin and by restrictive migration rules (Beck-Gernsheim 2007; Çelikaksoy et al. 2006; Timmerman et al. 2009).

Partner choice among migrants has been extensively studied, yet few studies in Europe exclusively focus on the descendants of these migrants, the so-called second generation. Our study aims to fill this gap. We do so by studying the second generation of the largest single migrant group in Europe: those of Turkish descent (Eurostat 2011). Furthermore, our study goes beyond earlier research on partner choice among migrants in several ways. First, while most studies only contrast endogamous unions and intermarriage, we explicitly include alternative partner choice options, i.e., an endogamous union with a second-generation partner of the same origin, and intermarriage with a partner of another migrant group. The latter two options constitute alternatives which become relevant in view of the ethnic diversification of European populations. The growing size of the second generation in marriageable ages in Europe translates into more opportunities to opt for an endogamous union in which social and cultural capital is shared between partners (Esser 2001; Esveldt et al. 1995; Lichter et al. 2011). The size of the ethnic group has been found to play an important role in the partner choices of first-generation migrants (Blau 1994; Lieberson and Waters 1988). In addition, a more diverse ethnic composition of the young adult population increases the opportunities of partnering with someone of another migrant origin. Nowadays, around 9 % of the EU population is foreign born. Individual countries’ estimates on migrants, their descendants and naturalized persons range between 13 % (e.g., Austria and Belgium) to 30 % (e.g., Switzerland) of population shares (Eurostat 2011).

Second, previous work focused on the role of families and ethnic communities on partner choice (Chiswick and Houseworth 2011; Kalmijn 1998), but did not examine the importance of engaging in social relations with members of other ethnic groups. We include a direct measure of exposure to cross-ethnic relations in this paper and study the role that peers, particularly those from different ethnic origins, have in partner choice. The importance of peers has hardly been studied, even though contact with peers—for instance, in a school setting—offers a direct opportunity to gain knowledge about and establish contacts with the majority as well as other ethnic groups. In addition, earlier US studies attest to the potential importance of peer contacts in which interethnic friendship during adolescence was also found to predict later intermarriage (Clark-Ibanez and Felmlee 2004; King and Harris 2007).

Finally, this study adds an innovative comparative dimension in which second-generation Turks across Europe are analyzed. Our study covers 13 cities in seven European countries with sizable groups of Turkish migrants. The 2007–2008 TIES (“The Integration of the European Second Generation”) survey collected information on second-generation respondents aged 18–35 years using a similar questionnaire and design in each city. This not only allows for comparison of partner choice patterns of one ethnic group across cities, but also the testing of the relevance of macro-level factors, such as local group size, migration, and integration policies. These latter policies potentially influence partner choice as they set different barriers for migration and stimulate contact with the majority population in distinct manners. The countries in our study—the Netherlands, Belgium, Sweden, Austria, Germany, France, and Switzerland—exemplify different migration and integration regimes. Comparing the same second-generation group across Europe provides important insights into the mechanisms behind partner choice of the growing second generation.

## Background

The Turkish are the largest single-origin group of migrants in Europe, with the largest share (defined as being born in Turkey or having at least one Turkish parent) living in Germany ((Statistisches Bundesamt 2007): 2,744,800 (2005)), followed by the Netherlands (Statistics Netherlands 2012: 373,000 (2007)), and France ((INSEE 2008): 222,000). During the 1960s and 1970s, Western European countries recruited “guest workers,” resulting in the influx of substantial numbers of migrants of Turkish descent. The majority of these migrants concentrated in old urban industrial areas. In general, these former Turkish labor migrants are characterized as having rural origins and low educational levels, and are reported as being part of cohesive communities that maintain strong ties to Turkey (Lievens 2000; Timmerman et al. 2009). Although the majority of the second generation are children from these original labor migrants, the composition of the Turkish community in European countries varies by ethnicity, religion, and region of origin. Clear dividing lines and limited social interaction exist between these groups (Wilpert and Gitmez 1987). Across Europe, the second-generation Turkish population, when compared to native populations, generally has lower levels of both educational and occupational attainment, as well as reduced prospects in the labor market (for an overview see Heath et al. 2008). At the same time, the second generation is experiencing upward social mobility compared to their parents (Heath et al. 2008).

Owing to their young age structure and the difficulty of identifying them in substantial numbers in surveys, our knowledge about the demographic behavior of second-generation Turks in Europe is limited. Existing studies on partner choice from the Netherlands and Belgium suggest that approximately two-thirds of the second generation marry a partner from Turkey. Marriage to another second-generation Turk seems to be less common (20–40 %), and intermarriage rates are below 10 % (De Graaf and Distelbrink 2005; Hartung et al. 2011; Lievens 1999; Reniers 2000; Timmerman et al. 2009). For Germany, however, it has been suggested that a growing proportion (up to 60 %) of second-generation Turks have a second-generation partner, although intermarriage rates remain low (Bentzin 1998; González-Ferrer 2006; Haug 2005). In addition, the number of marriage migrants from Turkey is declining in Germany, a trend which has also been observed in the Netherlands, but is much less prevalent in Belgium (Loozen et al. 2012; Ministerium für Generationen, Familie, Frauen und Integration 2008; Senatsverwaltung für Gesundheit und Soziales 1997; Timmerman et al. 2009).

## Determinants of Partner Choice

### Partner Choice Among the Second Generation

Many studies on intermarriage start from a framework in which three key factors—the role of third parties, personal preferences, and contextual constraints—influence whether people marry a partner from their own ethnic group or from a receiving country (Blau 1994; Kalmijn 1998; Lieberson and Waters 1988). However, the fact that an increasing proportion of people of Turkish origin in Europe belong to the second generation calls for an expansion of the number of available partner choice options. We argue that it is important to make a distinction between opting for a first-generation or a second-generation partner from Turkey within endogamous unions, as well as between opting for a native partner or a partner with another migrant background within exogamous unions.

Previous North-American studies that included the second generation suggested that the choice for a native partner reflects assimilation into the receiving country and indicates high levels of embeddedness into these societies and weakening roots with the culture of the ethnic community (Alba and Nee 2003; Gordon 1964; Klein 2001; Lieberson and Waters 1988). First-generation partners from the country of origin, on the other hand, would be valued out of a perceived preservation of traditional family values. Thus, the choice for a first-generation partner (or marriage migrant) is thought to reflect a wish for spouses who are not “spoiled” by Western lifestyles in Europe (Timmerman 2006). In addition, marrying a first-generation partner offers the possibility of maintaining close ties with family members still in Turkey (Beck-Gernsheim 2007; Çelikaksoy et al. 2006; DiCarlo 2007; Lievens 1999; Strassburger 2003).

If those who marry a first-generation partner are mainly oriented toward the country of origin and those who marry someone from the receiving country are mainly oriented toward the country of settlement, we expect the choice for a second-generation partner to be located somewhere halfway on this continuum. Those with a second-generation partner are likely oriented toward both the country of origin and the receiving country (in terms of contact and family values)—a characteristic that is shared by both partners (Lichter et al. 2011). Compared to an often arranged union with a first-generation partner (Reniers 2001), a second-generation partner may offer more opportunity for individual choice and romantic love match-making, but without necessarily crossing the boundaries between ethnic groups. A second-generation partner may incur less social conflict and social status loss within their own ethnic community than with choosing a native partner, particularly for women (Nauck 2001).

Crossing ethnic boundaries within a union may be a particularly difficult process among second-generation Turkish women, who in general are subject to higher levels of control than men (Phalet and Schönpflug 2001). In this sense, it can be expected that having a partner of another migrant origin largely resembles native partner choice. The propensity to opt for a partner of another migrant origin may also reflect the growing heterogeneous urban environments, where meeting people from other ethnic origins is increasingly likely (Blau 1994).

### Third Parties

Partner choice is hardly ever a completely individual or couple-based process, as individuals look for social approval and are susceptible to group norms. Groups offer identity, provide stability and uphold cultural norms. These functions can be expected to be particularly important among migrant groups. Strict partner choice endogamy can be seen as particularly advantageous by members of these groups, as it facilitates the preservation of group identity (Kalmijn 1998). Third parties are important in this process because they transmit values and norms during socialization (Biddle et al. 1980; Harris 1995; Newcomb and Bagwell 1995). Furthermore, third parties can act as role models; persons can observe behaviors of other third party members (social learning) and base their decisions on these experiences (Bongaarts and Watkins 1996; Montgomery and Casterline 1993). Thus, third parties are expected to exert a strong influence on the partner choice process. We focus on parents and non-coethnic peers, as they represent the two partially opposing socializing cultures to which second-generation Turks are exposed.

#### Parents

According to socialization theory, parents transmit values and norms to their children (Youniss and Smollar 1985). Parental values and attitudes are often assumed to strongly influence life course decisions among second-generation Turks, as the collectivistic nature of the Turkish culture highlights group interdependence, conformity to norms, and respect of older persons (Kagitçibasi 1996; Nauck 2002). Recent research nevertheless emphasizes that reality is more complex, and that different models of family values and attitudes co-exist in Turkey (Kagitçibasi and Ataca 2005). Next to the traditional family model of interdependence, a more recent family model of psychological interdependence can be identified.

Although conformity to parental preferences and expectations remains relatively strong, in the family model of psychological interdependence parents raise their children to be more autonomous in order for them to be more competitive in an urban environment (Kagitçibasi and Ataca 2005). In this family model, children have a greater say in life course decisions and couple-initiated love marriages are common (Hortaçsu and Oral 1994; Nauck 2001). Qualitative research among second-generation Turks in Europe has shown that this family model of psychological interdependence is also evident among Turkish migrant populations (Hooghiemstra 2001; Strassburger 2003). It is more common among parents with weak religious commitment, an urban origin, relatively small families, and high levels of human capital (Kagitçibasi and Ataca 2005; Koç 2008). These parents may also have higher educational and occupational aspirations for their children, and a weaker sense of attachment to the country of origin (Çelikaksoy et al. 2002).

In the traditional family model of interdependence, the emphasis on collectivism and dependence of children is still strong (Kagitçibasi and Ataca 2005; Koç 2008). Among families adhering to this model, marriages are often arranged by the parents or the extended family, and consanguineous unions are common (Koç 2008; Reniers 2001). In these families, the choice for a first-generation partner from Turkey is likely to be more common. Low levels of parental human capital, rural origin (particularly from Anatolian provinces (Hortaçsu and Oral 1994; Nauck 2002)), strong religious commitment, a gender-specific division of labor, and a large family size are characteristics associated with the traditional family model (Kagitçibasi and Ataca 2005). Thus, we expect that second-generation Turks whose parents have high levels of parental human capital, few children, no rural Anatolian origin, and did not raise their children religiously are more likely to have a native or second-generation partner and less likely to have a first-generation partner than second-generation Turks whose parents have the opposite set of characteristics (H1).

#### Peers

Peers constitute another influential “third party” in the partner choice process. For second-generation Turks, peers and particularly close friends are a primary contact to the majority population and other migrant groups outside the Turkish group. Because interethnic contact and knowledge increase feelings of cultural closeness (Pettigrew 1998), we expect that the presence of numerous non-Turkish persons within a peer network may to lead to higher rates of intermarriage (Alba and Golden 1986; Gordon 1964; Lieberson and Waters 1988; Pagnini and Morgan 1990). Contact with out-group members, here called non-coethnics, is most likely to develop in the school context, where adolescents spend most of their time and where minority and majority groups intermingle. Although adolescents are most likely to form friendships with those who hold similar characteristics, close contact in organized spheres, such as schools, may lead to more positive ethnic attitudes and more extensive interethnic interaction (Hallinan and Smith 1985), thus increasing the chances of developing close interethnic friendships or romance in adulthood (Vaquera and Kao 2008). Friendship with non-coethnics not only tends to increase feelings of cultural closeness and resemblance, but was also found to be connected to a higher desire for autonomy among Turkish students in Germany (Reinders and Varadi 2008). This type of friendship may thus increase the wish to participate more actively in partner choice decisions. Besides, intermarriage is likelier if an adolescent interacts with persons from other groups, as partners are often introduced via social networks. In addition, these interethnic networks can offer support to persons in an interethnic partnership (Clark-Ibanez and Felmlee 2004; King and Harris 2007). Thus, we expect that second-generation Turks who have more contact with non-coethnic peers, both as close friends and as more distant acquaintances, during secondary school are more likely to have a native or second-generation partner, while those with few contacts outside their own group are more likely to have a first-generation partner (H2).

### Individual Characteristics

The preference for a partner with similar characteristics is widespread. In general, ethnic (Jones and Luijkx 1996), cultural, and religious (Hendrickx 1994; Foner and Alba 2008) homogamy is the most common. However, in some circumstances, other characteristics may become more important than ethnic ones. For example, migrants with high levels of education have been found to be more willing to exchange ethnic traits for a partner with similar levels of education (Furtado 2006; Kalmijn 1993; Lievens 1998). Furthermore, their adaptation to customs of the receiving society is easier and by leaving ethnic enclaves, highly educated individuals will have more opportunities to establish contact with potential spouses of other ethnicities (González-Ferrer 2006; Kalmijn 1998; Qian and Lichter 2001). In other words, for second-generation Turks, higher levels of education may lead to a stronger preference for autonomy in life course choices. However, findings on the educational effect among the Turkish second generation are inconclusive. Lievens (1999) found, generally, that low educated men and higher educated women choose a first-generation partner. However, the latter finding was not supported by other studies (Hooghiemstra 2001; González-Ferrer 2006).

Another factor influencing partner choice is the birth cohort. Recent cohorts are more likely to out-marry (Joyner and Kao 2005). The younger a cohort is, the longer the particular migrant group has resided in the receiving society, potentially resulting in reduced social distance between minority and majority group and increased assimilation into mainstream society. The shared experience of living in one country may blur boundaries and make intermarriage across ethnic groups more acceptable. In addition, contacts to the parents’ country of origin may be weaker, the younger the cohort is. In growing up and interacting in the receiving country, the second generation may gain more influence over their own life course decisions, particularly as these changes likewise occur in Turkey (Hooghiemstra 2001; Strassburger 2003). We hypothesize that second-generation Turks belonging to a younger birth cohort and with higher levels of education are more likely to opt for a native or second-generation partner and less likely to opt for a first-generation partner than those belonging to an older birth cohort and with lower levels of education (H3).

### Contextual Factors

Contextual factors influence partner choice by defining the opportunities and constraints for meeting potential partners. Individuals search for partners with similar characteristics to themselves in the local marriage market. In case of a shortage of suitable persons, people need to be more flexible in their preferences to find a partner. Among the best known context factors to affect partner choice of migrants is the size of migrant communities (Blau 1994; Klein 2001; Lieberson and Waters 1988). The larger the group, the more likely a person will encounter a suitable partner from his/her own ethnic group (Chiswick and Houseworth 2008; González-Ferrer 2006; Lieberson and Waters 1988; Lievens 1998). A larger group may also provide more opportunities for the enforcement of in-group rules, such as endogamy. As previously indicated, the Turkish are the largest single migrant origin group in Europe. Although they are scattered across Europe, within regions they are geographically concentrated, which facilitates intra-group contact. Levels of local concentration nevertheless differ, leading to varying chances of finding a suitable partner among the second generation of Turkish origin. We expect that the higher the relative number of second-generation Turks of marriageable age in a city is, the more likely it is for Turkish second generation young adults to have a second-generation partner rather than a first-generation or native partner (H4).

Another contextual factor that likely influences partner choice of the second generation is the migration and integration policies of the receiving country. Restrictive migration policies in Europe provided incentives for a continuous influx of spouses from the country of origin, as marriage migration was the only way to enter Europe after the end of labor recruitment in the early seventies. Classically, three types of integration policy models are distinguished (Brubaker 1992; Castles and Miller 2003): differential exclusion, assimilation, and multiculturalism. The Migration Integration Policy Index (MIPEX)—that includes information on over 100 indicators covering minority integration policies in various areas (Niessen et al. 2007)—shows that migration policies across Europe still mirror this typology quite closely. Countries thought to represent the multiculturalist model, such as Sweden, Belgium, and the Netherlands, provide the most generous procedures for migrants and family members to enter a country and grant them political rights, access to the labor market, security of residence, state support, and possibilities to uphold cultural norms. Bringing in a partner from the country of origin is easier in these countries compared to countries with more restrictive policies, such as France, Germany, and Switzerland. In these latter countries, which fall into the differential exclusionist and assimilation model, barriers to becoming a citizen and being granted support are higher. Austria gives the least rights to migrants in Europe, as far as access to the labor market and possibilities of family reunion are concerned. In these last two groups of countries, it will be more difficult to marry a first-generation partner. Based on these arguments, it is expected that in countries such as the Netherlands, Belgium, and Sweden—with traditionally less restrictive migration and integration policies—second-generation Turks are more likely to have a first-generation partner than in more restrictive-integration-policy countries such as France, Germany, Austria, and Switzerland (H5).

## Data and Method

### Data

We analyze data from “The Integration of the European Second Generation” (TIES 2007–2008) survey.^1^ TIES is the first large-scale European comparative survey focusing exclusively on the second generation. Around 10,000 descendants of migrants from Turkey, Morocco, and the former Yugoslavia along with a native comparison group aged 18–35 years participated in face-to-face interviews in 15 cities in eight European countries. An identical questionnaire was used in all cities covering different domains of life; but so far, to our knowledge, no cross-national comparative study on intermarriage has been published on the data (for related national studies see: De Valk 2008; Hartung et al. 2011; Huschek et al. 2011; Milewski and Hamel 2010).

Respondents were sampled as second-generation Turks if they were born in the country where the survey was held and at least one of their parents was born in Turkey.^2^ In the Netherlands, Sweden, and Belgium, the sample frames were the population registers. For France, Germany, Austria, and Switzerland, onomastic techniques were used to identify members of the Turkish community. This method was chosen because in France, only information on an individual’s birth country, and not that of their parents, was available, and in the German-speaking countries, strict data protection laws prevented access to population register data. Owing to the absence of a comparable sample frame across countries, it was impossible to have a representative survey. Furthermore, the overall response rate varies between 24 and 50 % per city (Groenewold and Lessard-Phillips 2012). Additional non-response analyses that we carried out revealed that, overall, men and lower educated individuals were slightly underrepresented among all respondents. These findings are by and large comparable with other migrant surveys. Ethnic minorities generally have low response rates in many European countries (e.g., Feskens et al. 2006).

Despite their limitations, the data also has advantages. Given the current small numbers of second-generation individuals in the total population, a general sample would fail to yield enough respondents. A purposive sample is thus needed to reach meaningful sample sizes. This is also what makes the TIES data unique, as most existing European datasets have too few young adults of migrant descent to fully capture their life course decisions. And although registers in some of the Nordic countries have detailed information on the complete second generation in the population, they generally do not have detailed information on their parents and peer networks that allow for an understanding of the relevant mechanisms behind partner choice.

Our pooled sample contains data from 13 cities. For the analysis, we include all second-generation Turks who have (had) a first partner, resulting in a total sample of 1,437 respondents (800 women, 637 men). The cities included in our study are Amsterdam and Rotterdam (the Netherlands), Brussels and Antwerp (Belgium), Stockholm (Sweden), Paris and Strasbourg (France), Berlin and Frankfurt (Germany), Zurich and Basel (Switzerland), and Vienna and Linz (Austria).^3^ In order to reduce complexity and costs, data collection was limited to two cities per country in which a substantial share of Turkish second generation live. An urban sample frame was chosen because most migrants and their descendants live in urban areas. In the Netherlands, for example, 32 % of second-generation Turks live in Amsterdam and Rotterdam (Statistics Netherlands 2012). Likewise, in Sweden, Turks are mainly concentrated in urban areas, with over 50 % living in the Stockholm region (Westin 2003). In Germany, 61 % of the Turks live in cities with more than 500,000 inhabitants (Bundesministerium für Arbeit und Sozialordnung 2002). Similar patterns are found in all countries in our study (Herzog-Punzenberger 2003; Milewski and Hamel 2010; Timmerman et al. 2003; Wanner 2004).

### Variables

#### Dependent Variable

Our dependent variable is a categorical variable indicating the ethnic background of the first partner with whom our second-generation Turkish respondents live(d). Five partner types were distinguished based on the questions about the country in which the partner, his/her mother and father where born as well as the age of migration of the partner (Table 1). A first-generation partner (Type 1) is defined as a person who was born in Turkey and came to Europe after 6 years of age. 92 % of these partners migrated after the age of 17 years. A second-generation partner (Type 2) is defined as being born outside Turkey to at least one Turkish parent, or as being born in Turkey but having migrated before the age of 6 years and thus being socialized mainly in Europe. A native partner (Type 3) is characterized by having parents who were born in one of the seven countries in our study. Finally, a first-generation (Type 4a) and a second-generation partner (Type 4b) from a different migrant group are defined like Types 1 and 2, but originating from countries other than Turkey and the receiving countries. Types 4a and 4b are relatively uncommon, particularly a first-generation partner of other migrant origin, therefore we combine these two partner types in the analysis. Because the proportion of respondents with a partner from another migrant group was relatively small and because the ethnic origin of these partners was quite heterogeneous, it was decided to only present descriptive information on having a migrant partner of another origin, and not to include this type into the multivariate analyses.

**Table 1:** A classification of partner types

	Partner type	Partner’s country of birth	Migration age	Partner’s parents country of birth
1	First generation, same origin	Turkey	>6 years	Turkey
2	Second generation, same origin	Turkey	≤6 years	Turkey
		Receiving country^a^		Turkey
3	Native	Receiving country^a^		Receiving country
4a	First generation, other migrant origin	Any other country	>6 years	Any other country
4b	Second generation, other migrant origin	Any other country	≤6 years	Any other country
		Receiving country^a^		Any other country

^a^Either Sweden, the Netherlands, Belgium, France, Germany, Switzerland, or Austria

#### Independent Variables

The selection of independent variables (Table 2) to be used in the multivariate analysis is limited to those for which information on the period prior to the main union formation years is available. To avoid problems of reverse causality, attitudinal variables and other variables that relate to the time of the interview are excluded from our analysis, as these variables may have changed as a result of union formation experiences.

**Table 2:** Descriptive information on independent variables by country (mean and standard deviation)

		Total		Netherlands		Belgium		Sweden		France		Germany		Switzerland		Austria	
	*N*	1,437		226		296		135		167		225		178		210	
	Range	Mean	SD	Mean	SD	Mean	SD	Mean	SD	Mean	SD	Mean	SD	Mean	SD	Mean	SD
Family factors																	
Human capital mother	−2.25 to 1.40	−0.26	1.00	−0.38	1.05	−0.49	1.06	0.29	0.90	−0.47	1.00	−0.58	1.08	0.09	0.90	0.14	0.87
Human capital father	−4.53 to 1.05	−0.19	1.10	−0.35	1.28	−0.37	1.27	0.27	0.52	−0.39	0.80	−0.36	1.11	−0.03	0.99	0.22	0.98
Parents grew up in rural Anatolia	0/1	0.51	0.50	0.64	0.48	0.41	0.41	0.75	0.43	0.43	0.50	0.53	0.50	0.40	0.49	0.45	0.50
Number of siblings	0–6	3.01	1.44	3.13	1.51	3.46	1.50	3.09	1.45	2.98	1.53	2.95	1.31	2.47	1.30	2.67	1.21
Peer factors																	
Contact to non-coethnic peers	−3.00 to 1.14	−0.15	1.05	−0.26	0.84	−0.22	1.06	−0.08	1.16	0.20	0.88	−0.42	1.13	0.15	1.09	−0.15	1.03
Percentage of natives in secondary school	1–5	3.35	1.07	2.89	1.18	3.19	1.00	3.35	1.04	3.32	0.96	3.4	0.80	3.67	1.12	3.81	1.07
Individual characteristics																	
Birth cohort																	
1970–1974	0/1	0.17	0.38	0.17	0.38	0.14	0.35	0.13	0.34	0.13	0.33	0.24	0.43	0.23	0.42	0.15	0.36
1975–1979	0/1	0.40	0.49	0.42	0.49	0.46	0.50	0.47	0.50	0.49	0.50	0.39	0.49	0.29	0.46	0.26	0.44
1980–84	0/1	0.34	0.47	0.36	0.48	0.28	0.45	0.35	0.48	0.32	0.47	0.32	0.47	0.33	0.47	0.43	0.50
1985–1990	0/1	0.10	0.29	0.05	0.22	0.11	0.32	0.05	0.22	0.07	0.25	0.05	0.23	0.15	0.35	0.16	0.37
No completed level secondary education	0/1	0.07	0.25	0.16	0.37	0.03	0.18	0.02	0.12	0.07	0.26	0.03	0.17	0.16	0.37	0.02	0.15
Lower secondary education	0/1	0.45	0.50	0.64	0.48	0.49	0.50	0.24	0.43	0.54	0.50	0.36	0.48	0.60	0.49	0.21	0.41
Vocational track	0/1	0.30	0.46	0.13	0.34	0.25	0.44	0.42	0.49	0.18	0.39	0.50	0.50	0.16	0.37	0.46	0.50
General/academic track	0/1	0.19	0.39	0.075	0.26	0.22	0.42	0.33	0.47	0.21	0.41	0.12	0.32	0.09	0.29	0.31	0.46
Context factors																	
Size Turkish second generation 18–35 years	0.14–1.40	0.73	0.32	1.11	0.31	0.89	0.04	0.76	0.00	0.42	0.13	0.88	0.15	0.44	0.07	0.32	0.16
Multicultural immigration policies	0/1	0.46	0.50	1.00	0.00	1.00	0.00	1.00	0.00	0.00	0.00	0.00	0.00	0.00	0.00	0.00	0.00
Control variables																	
Not raised religiously	0/1	0.16	0.36	0.12	0.33	0.13	0.34	0.39	0.49	0.17	0.38	0.08	0.28	0.29	0.45	0.08	0.27
Christian	0/1	0.29	0.17	0.02	0.14	0.02	0.12	0.16	0.37	0.02	0.14	0.03	0.16	0.01	0.12	0.01	0.10
Shia/Alevi	0/1	0.10	0.30	0.07	0.26	0.06	0.24	0.03	0.16	0.06	0.23	0.23	0.47	0.17	0.38	0.10	0.30
Sunni	0/1	0.71	0.45	0.79	0.41	0.80	0.40	0.43	0.50	0.75	0.43	0.66	0.47	0.53	0.38	0.81	0.39
Woman	0/1	0.58	0.50	0.54	0.50	0.47	0.50	0.54	0.50	0.70	0.46	0.57	0.50	0.54	0.50	0.60	0.49
Age at union formation	15–34	21.94	3.10	21.67	2.96	21.61	3.28	22.32	3.00	22.12	3.24	22.94	3.11	22.22	3.09	21.02	2.84

*Source* TIES data 2007–2008

Four indicators of parental characteristics are constructed. The level of human capital of the mother and of the father are two factor scores which were calculated using principal-component factor analysis, and were based on the following variables for mothers and fathers separately: educational level of mother and father (no = 1, low = 2, medium = 3, high = 4), literacy of mother and father (no = 0, yes = 1), mother and father’s knowledge of the language of the receiving country (no = 0, read = 1, read and write = 2), and mother having paid work when the respondent was 15 years of age (no = 0, yes = 1). The higher the factor score, the higher the level of parental human capital. Parent grew up in rural Anatolia is a dummy variable indicating whether or not the mother or the father had mainly lived in a rural area in an Anatolian province before they were 15 years-old (0 = not from rural Anatolia, 1 = from rural Anatolia). Usually, both parents come from a similar region (90 %). The variable is used as a proxy for traditional parental behavior and attitudes (Nauck 2002). It was constructed with variables indicating from which province the parents came and whether or not the parent lived in a city in Turkey. The final characteristic of the parental home is the number of siblings of respondents.

Two indicators measure contacts to non-coethnic peers in secondary school. Contact to non-coethnic peers is a factor score constructed from the variables “ethnicity of best friend” (own ethnic group = 1, other ethnic group = 2, native group = 3) and the dummy variable “Natives in wider friend network” (no = 0, yes = 1). An increasing factor score means a higher level of contacts to non-coethnic peers. Percentage of natives in secondary school measures the ethnic composition of the secondary school attended by the respondent. Respondents reported whether their school had almost no native students (=1), up to 25 % (=2), approximately 50 % (=3), up to 75 % (=4), or almost all native students (=5). The models include a squared term of the variable to assess non-linearity.

Two variables refer to individual characteristics. Cohort changes in partner choice are captured by the inclusion of dummies for 5 year birth cohorts (1970–1974, 1975–1979, 1980–1984, 1985–1990). The oldest cohort (1970–1974) constitutes the reference category. The highest level of secondary education completed is measured by a set of dummy variables, distinguishing respondents who have either no completed degree in secondary education or a special education degree (=1), a lower secondary education degree (=2), a degree of higher secondary education in a vocational track (=3) or a degree in a general or academic higher secondary level (=4). This last group of respondents is used as the reference category.

Two variables are used to examine the role of context. The size of the second-generation Turkish population within the 18–35 years age group is a city-level variable constructed by dividing the approximate number of the second-generation Turks within the 18–35 age group (TIES target age group) by the number of inhabitants per city. This number gives an approximation of the size of the Turkish population in the age-range in which most marriages occur. The city level was chosen because most marriage markets are local in scope (Lichter et al. 1991). Multicultural immigration policies is a dummy variable created to distinguish countries characterized by multicultural integration policies that have on average less restrictive immigration and integration policies from countries described by the differential exclusion and assimilation models. The latter countries, to a greater extent, limit family reunification, according to the MIPEX scale. Belgium, the Netherlands, and Sweden are coded as having less restrictive immigration policies, the other countries as having stricter policies.

In addition, the models include several control variables, such as gender. Age at union formation is included because arranged marriages often occur at an earlier age than couple-initiated marriages (Fox 1975; Nauck 2001). Religious upbringing is used to control for within-group differences among the Turkish second generation. Furthermore, choosing a partner with the same religion is usually preferred and may influence partner choice. Respondents stated whether they grew up without religious upbringing (1), or according to Christian (2), Sunni (3), or Shia/Alevi (4) faith. Dummies were created for each of these religious groups with Sunni—the most common religious upbringing—being the reference category.

A series of dummy variables were created to distinguish the seven countries in our study: Sweden, the Netherlands, Belgium, France, Germany, Switzerland, and Austria.

In the descriptive part, we also present information on who took the initiative in finding a partner and whether the partner was a relative as indicators of the predominant marriage system (Hortaçsu and Oral 1994; Nauck 2001). “Initiative” is a self-reported dummy variable in which couple-initiated (meeting through friends, in school or university, at work, while going out, in a public place or during a holiday) and family-initiated (meeting through family introduction, parents’ network or holiday in parents’ home country) ways of meeting each other are juxtaposed. “Consanguineous union” is a dummy registering whether or not the partner is a relative. We include these variables in our descriptive analysis for the sole reason that they are endogenous to the particular partner choice process. In addition, both questions were not asked in Belgium and Sweden.

### Methods

First, we present descriptive results on partner choice by country, also taking into account who took the initiative and whether the partner was a relative or not. Second, we present multivariate analyses in which we test the hypotheses formulated in the background section. Two main options for analyzing these data were available. First, a competing risk hazard model with the timing of entry into a union as the dependent variable and the four types of outcomes (a union with a first-generation partner, a union with a second-generation partner, a union with a native, and no union) was estimated. This approach also includes individuals who have not yet entered a union. A main advantage of this analytical approach is that it allows a correction for censoring. However, the drawback is that it is rather complicated to interpret the actual partner choices differences we are primarily interested in, as this requires a comparison of rates across destinations and determining whether they are significantly different from one another. Although feasible, this is a rather cumbersome procedure. Second, we ran multinomial logistic models with the three partner types as outcomes and to directly compare odds ratios across destinations. Since results of both sets of analyses were highly similar, we decided to present the results of the multinomial logistic models, as these are somewhat easier to interpret for the purpose of our study (results from competing risk hazard models are available from the first author on request).

Because results for men and women were highly similar, we pooled data for both sexes and only include interactions if necessary. Given the small number of higher-order units (seven countries or 13 cities), we refrain from estimating multilevel models, but correct for intragroup correlations at the country level by using robust standard errors. As a result, the tests of the hypotheses on context effects can only be viewed as tentative ones.

## Results

### Partner Choice by Country

Table 3 shows the distribution of partner choices by country.^4^ With the exception of Germany, second-generation Turks are more likely to opt for a first-generation partner. The percentages vary between 41 and 64 % for men and between 45 and 79 % for women. In Germany, only 13–14 % of the respondents have a first-generation partner, but 65 % of men and 71 % of women have a second-generation partner. In the other countries, 17–30 % of men and 16–27 % of women have a second-generation partner. Intermarriage rates with a native partner vary between 11 and 25 % for men and 1 and 17 % for women. The proportions of second-generation Turks with a partner from another migrant group (either first or second generation) are quite small, with the exception of male respondents in Switzerland (23 %) and Sweden (19 %). Second-generation women in Belgium, the Netherlands, and Switzerland report higher intermarriage rates with other migrant partners than with native partners. Overall, men are less likely than women to have a first-generation partner from Turkey, and more likely to have a native or other migrant partner.

**Table 3:** Partner type in percent among second-generation Turks by country and gender

Country of residence	From Turkey		Native	Other migrant origin		*N*
	First-generation partner	Second-generation partner				
Sweden						
Men	40.6	21.9	18.8	18.8	100.0	64
Women	45.1	25.4	16.9	12.7	100.0	71
The Netherlands						
Men	53.9	29.7	11.0	5.5	100.0	91
Women	67.4	25.9	2.2	4.4	100.0	135
Belgium						
Men	63.5	21.8	12.2	2.5	100.0	156
Women	79.3	16.4	1.4	2.9	100.0	140
France						
Men	44.2	23.1	25.0	7.7	100.0	52
Women	70.4	16.5	9.6	3.5	100.0	115
Germany						
Men	13.3	65.3	18.4	3.1	100.0	98
Women	14.2	70.9	13.4	1.6	100.0	127
Switzerland						
Men	46.0	17.2	13.8	23.0	100.0	87
Women	61.5	22.0	3.3	13.2	100.0	91
Austria						
Men	56.2	21.4	12.4	10.1	100.0	89
Women	61.2	26.5	9.9	2.5	100.0	121
Total						
Men	47.1	29.0	14.9	9.0	100.0	637
Women	57.9	29.6	7.5	5.0	100.0	800

The differences in partner choice between cities^5^ in the same country are relatively small, particularly among women. The main exception are second-generation Turkish men living in Brussels. They are less likely to have a first-generation partner and are more likely to have a native partner than those in Antwerp. Another difference is found for Turkish second-generation men in Paris. They are more likely than those in Strasbourg to have a partner of other migrant origin.

### Partner Choice by Initiative and Consanguineous Union

In Table 4, information is given on who took the initiative in the partner selection and on the role played by consanguineous marriage. The patterns are almost identical for men and women and are thus presented jointly. The two variables show similar results by partner type. Second-generation Turks with a native partner or a partner of other migrant origin met mainly in couple-initiated ways. There is, additionally, a clear difference between those who have a first- or a second-generation partner from Turkey: one-third of those with a second-generation partner met in a family-initiated way, while two-thirds met a second-generation partner in a couple-initiated manner. For those with a first-generation partner from Turkey, it is the other way around. Consanguineous marriages are more common among those with a first-generation partner from Turkey. Taken together, these findings suggest that the traditional marriage system is more applicable to second-generation Turks with a first-generation partner than to those with a second-generation partner and even less to those with a native partner and a partner of another migrant origin.

**Table 4:** The role of initiative and consanguineous union in partner choice

	From Turkey		Native	Other migrant origin
	First-generation partner	Second-generation partner		
Initiative				
Family-initiated	65.1	33.9	4.8	11.1
Couple-initiated	34.9	66.1	95.2	88.9
	100.0	100.0	100.0	100.0
*N*	430	295	62	36
Consanguineous partner				
No	73.7	89.7	100.0	100.0
Yes	26.3	10.3	0.0	0.0
	100.0	100.0	100.0	100.0
*N*	449	290	46	31

Information is only available for those whose current partner is their first partner and for respondents not living in Belgium or Sweden

### Multivariate Results of Partner Choice

In Table 5, the results of the multinomial logistic regressions testing our hypotheses on partner choice among second-generation Turks are presented. In Model 1, the variables testing hypotheses 1–3 are discussed. Model 2 introduces the context variables testing hypotheses 4 and 5.

**Table 5:** Multinomial logistic regression estimates (odds ratios) of partner choice among second-generation Turks

First-generation partner is base outcome	Model 1		Model 2	
	2nd generation	Native	2nd generation	Native
Family factors				
Human capital mother				
Factor score	1.22*	1.44**	1.19*	1.36**
Human capital father				
Factor score	1.14^#^	1.52**	1.11	1.41**
Parents grew up in rural Anatolia	0.83	0.64*	0.90	0.71*
Number of siblings	0.99	0.84*	0.98	0.84**
Peer factors				
Contact to non-coethnic peers				
*Factor score*	0.88^#^	1.60***	0.82*	1.53*
Percentage natives secondary school	1.49	0.46	1.75	0.62
(Percentage natives secondary school)^2^				
*Squared variable*	0.95	1.12	0.92	1.05
Individual characteristics				
Cohort				
1970–1974	1.00	1.00	1.00	1.00
1975–1979	1.58*	0.61^#^	1.52*	0.62
1980–1984	2.64***	0.76	2.28***	0.69
1985–1990	2.46**	0.44	2.16*	0.39*
Completed level secondary education				
No secondary education	0.61	0.26*	0.43**	0.16
Lower secondary degree	0.62*	0.48*	0.55**	0.38**
Vocational track	0.77	0.81	0.82	0.82
General/academic track	1.00	1.00	1.00	1.00
Context variables				
Country				
Sweden	1.00	1.00		
The Netherlands	1.39	0.65		
Belgium	0.69	0.58		
France	0.86	1.53		
Germany	15.7***	8.86***		
Switzerland	0.93	0.55		
Austria	0.78	0.64		
Size Turkish second generation 18–35 years			15.53*	5.17
Multicultural immigration policies			0.16**	0.25*
Control variables				
Man	1.00	1.00	1.00	1.00
Woman	0.13^#^	0.01**	0.17**	0.01**
Age union formation	1.01	0.96	1.03	0.98
Age union formation *Woman	1.08^#^	1.16*	1.07*	1.16*
Religious upbringing				
No religious upbringing	0.92	2.29**	1.05	2.40*
Christian	4.76**	15.03***	6.90***	20.16***
Shia	1.08	2.52**	1.43	2.64*
Sunna	1.00	1.00	1.00	1.00
Observations	1,340		1,340	
Log likelihood	−980.5		−1,030.8	
*R* ^2^	0.21		0.17	

^#^ *p* < 0.10; * *p* < 0.05; ** *p* < 0.01; *** *p* < 0.001

#### Third Parties: Parents and Peers

We find clear effects of third parties (parents and peers) on the different partner choice outcomes. Table 5 shows that the relative chances of having a second-generation partner rather than a first-generation partner increase with an increase in mother’s human capital. The same is true for the human capital of the father, but only at the *p* < 0.10 level. A high human capital of the mother and the father also significantly increases the relative likelihood of having a native partner compared with a first-generation partner, while a larger number of siblings and having parents originating from rural Anatolia reduces the likelihood of having a native partner. Thus, the findings for parental characteristics are largely in line with hypothesis 1.

Focusing on the influence of persons outside the family network and ethnic group, we find that having more contact to non-coethnic peers increases the likelihood of having a native partner. These findings are in line with hypothesis 2. However, we expected that having contact with non-coethnic peers would increase the likelihood of having a second-generation partner as well. The opposite, however, has turned out to be true. Contrary to our expectations, having fewer non-coethnic contacts increases the likelihood of having a second-generation partner from Turkey. In Model 1, this is only significant at the *p* < 0.10 level, while in Model 2, the addition of context variables make this likelihood significant throughout. The influence of more distant acquaintances at school seems to matter less than close friends, as the estimates of the former are not statistically significant. This is true if a linear relationship is tested (results not shown) and if a U-shaped relationship is tested like is done in Table 5. Thus, support for hypothesis 2 seems limited to the influence of relatively close friends.

#### Individual Characteristics

Hypothesis 3 suggests that second-generation young adults with characteristics that predispose them to more modern attitudes are more likely to opt for a second-generation or a native partner. Although the effects for cohort are not completely linear, we generally find that the likelihood of having a second-generation partner from Turkey, rather than a first-generation partner, is higher among younger birth cohorts (Table 5). By contrast, younger cohorts are not more likely to have a native partner. In addition, there are clear educational differences in partner choice patterns, with respondents with only lower secondary education being less likely to opt for a second-generation partner than those who followed an academic track. For native partner choice, higher levels of secondary education are associated with a higher likelihood of having a native partner. No significant interaction effects between sex and secondary education level were found. These results partially support Hypothesis 3.

#### Context

Effects of the country variables are presented in Model 1 of Table 5. Country differences in partner choice are relatively limited when controlling for individual-level variables. However, second-generation Turkish individuals in Germany are still more likely than those in other countries to have a second-generation or native partner rather than a first-generation partner.

In Model 2 of Table 5, the country dummies are substituted by contextual variables in order to examine Hypotheses 4 and 5. In line with these hypotheses, we find that the size of the Turkish second generation in the 18–35 years age-group and the endorsement of multicultural immigration policies clearly influence the choice of a first-generation or second-generation partner from Turkey, but do not influence the choice of a native partner. In countries with predominantly multicultural integration policies and less strict immigration policies, second-generation Turkish men and women are more likely to choose a first-generation partner and less likely to choose a second-generation partner. In addition, we find that the higher the percentage of second-generation Turks of marriageable ages is, the higher the probability of choosing a second-generation partner and the lower the probability of selecting a first-generation partner. Because the largest share of second-generation partners are chosen by the Turkish descendents in Germany, we also estimated Model 2 excluding the German sample. Although the direction of the effects remain the same, the findings for the size of the young second-generation Turks per city are only significant at the *p* < 0.10 level, and the previous found effect for policy context is no longer significant.

#### Control Variables

The control variables show that the likelihood of having a second-generation or native partner is lower for women than for men. While there are no significant timing differences regarding union formation for men or women who have a second-generation or native partner are more likely to be older than those women who have a first-generation partner. Second-generation Turks without religious upbringing and of Christian or Shia/Alevi faith are more likely to have a native partner compared to a first-generation partner from Turkey. Similarly, second-generation Turks with a Christian religion are more likely to have a second-generation partner.

## Discussion and Conclusion

The objective of this paper was to amplify research on partner choice among the Turkish second generation by including the choice for a second-generation partner from Turkey as well as a partner of other migrant origin as important alternatives to having either a first-generation partner from the country of origin or a native partner. Next to studying the effects of parents and context, we included a measure of exposure to cross-ethnic relations through peers which takes into account the special situation of the second generation as being in between two cultures. This paper also provided a first comparative picture of partner choice among the Turkish second generation in Europe.

We found support for our first hypothesis, that parental characteristics matter, as indicators of the different family models existing among the Turkish second generation. Turkish young adults whose parents had characteristics that predispose them to the traditional family model (Kagitçibasi and Ataca 2005), where parental involvement and traditional family attitudes are more common, were less likely to enter a union with a second-generation partner from Turkey or with a native partner than were young adults whose parents had characteristics that predispose them to the more “modern” family model of psychological interdependence.

The results only partially support our second hypothesis on the role of peers. We found that contact with non-coethnic close friends during adolescence increased the likelihood of having a native partner, but also slightly decreased the likelihood of having a second-generation rather than a first-generation partner. A potential explanation for this finding is that one’s own group of friends is an important recruiting ground for a second-generation partner. This interpretation was supported by the descriptive findings showing that those with a second-generation partner mainly met their partner in couple-initiated manners. Therefore, contact with non-coethnic peers may not only constitute a measure of orientation toward the receiving country, but could also be indicative of meeting opportunities. Another important finding is that the composition of the school population during adolescence is not important. Thus, strong ties to non-coethnic peers, rather than weak ties, seem to play a key role in navigating important life-course decisions such as partner choice.

At the level of individual characteristics, we hypothesized that younger cohorts and those with a higher level of education would be more likely to develop autonomy preferences, thus making it more likely for them to take an active role in the union formation process and as a result, choose a second-generation or native partner. We found support for this hypothesis. Higher levels of completed secondary education increased the likelihood of the second generation choosing a native over a first-generation partner. For birth cohort, we found support for our hypothesis that younger cohorts would be more likely to choose a second-generation partner. The cohort effect may reflect a shift in preference from a first-generation to a second-generation partner within the younger cohorts. It may also reflect a size effect because more second-generation partners (and individuals from other migrant communities) are available in the local marriage market. However, we also found that younger cohorts were less likely to enter a union with a native. We believe this reflects a timing factor: those with native partners are usually the latest to enter a union. Many of the respondents from the youngest cohorts have not yet completed their union formation process, and may opt for a native partner in the future.

Context also plays an important role in partner choice outcomes. The descriptive overview of partner choice showed that the Turkish second generation overwhelmingly preferred first-generation partners from their parents’ country of origin. Only in Germany (Berlin and Frankfurt) did the majority of second-generation Turks opt for a partner from their own second generation. It is possible that the very large number of Turkish descendants in Germany compared to the other countries may play a role here. A second-generation partner represents the second or even third preferred option in the other countries. While, in general, rather similar partner choice outcomes could be observed in the different countries, multivariate analysis suggests that context factors influenced the choice between the first- and second-generation partners. The findings indicated that part of the preference for first-generation partners was connected to low numbers of Turkish second-generation youths at the city level and access to welfare state provisions and policies that promote multicultural integration, allowing more cultural expression and easier family reunification. This overall supported hypotheses 4 and 5, although the findings remain tentative and need to be tested further, as we could not test it rigorously.

One of the main contributions of our study is the simultaneous focus on multiple partner choice options among the Turkish second generation. Our results support the relevance of doing so, as the choice for a second-generation partner differs significantly from the choice for a first-generation partner. It is important to include the distinction between a first- and a second-generation partner from the sending countries in future studies on partner choice and to examine how the relative popularity of these two options varies in time and space. In addition, it is important to study the role of choosing a partner from another migrant group. In the current study, too few respondents opted for a partner from another migrant group, and those who did made very heterogeneous choices. Register data could be a useful source to further examine this specific partner choice option.

For the near future, we expect that a first-generation partner from Turkey will remain the most common choice among the Turkish second generation in Europe, as this was observed in 11 out of 13 cities in our study. As we have observed, a shortage of suitable second-generation partners does not increase intermarriage rates, but rather leads to an increased pressure to find a suitable first-generation partner (and to a lesser extent a partner of other migrant origin for men). Choosing a partner from the country of origin could be advantageous with respect to status and quality of the partner, particularly for the majority of young adults with a relatively low level of education. Furthermore, migration pressure, obligations to kin, and continued pressure by parents helps to explain why second-generation exogamous rates currently remain low (Bisin and Verdier 2000).

However, the choice for a first-generation partner from Turkey will become less common in the long run. Already, the combined percentages of second-generation Turks having a second-generation partner from Turkey, a native partner or a partner of other migrant origin are larger than those choosing a first-generation partner among men in all cities except those in Austria and the Netherlands. This is less the case for women. Moreover, the numbers of first-generation partners are likely to further decrease the larger the size of the Turkish second-generation population and the higher their levels of education become. Furthermore, as the Turkish community becomes more firmly rooted in their receiving countries, an increasing proportion of families will have relatively little contact with their family of origin. This will most likely reduce the pressure to arrange a marriage with a first-generation partner from Turkey. In addition, the second generation can be expected to gain increasing influence in the partner selection process (Hooghiemstra 2001; Strassburger 2003) for instance, due to the spread of the family model of psychological interdependence (Kagitçibasi and Ataca 2005; Koç 2008). As a result, romantic matches may become more common, and this requires that the couple meet each other before the marriage. This is likelier to happen with a local partner pointing to the importance of further studies on the local context and friendship networks for partner choice among the children of immigrants in Europe.

## Footnotes

1
www.tiesproject.eu.

2 Intermarriage among the parental generation is lower than 5 %.

3 Madrid and Barcelona were excluded, because no second-generation Turks were interviewed.

4 In order to simplify the argumentation, we speak about countries, although our data only represent on average two cities per country.

5 City level results are not shown.
